# New Onset Non-Convulsive Status Epilepticus Despite Cefepime Renal Dose Adjustment

**DOI:** 10.7759/cureus.12689

**Published:** 2021-01-13

**Authors:** Mosunmoluwa Oyenuga, Abayomi Oyenuga, Abdul Rauf, Omotola Balogun, Niranjan Singh

**Affiliations:** 1 Internal Medicine, SSM Health St. Mary's Hospital, St. Louis, USA; 2 Internal Medicine, University of Minnesota School of Medicine, Minneapolis, USA; 3 Internal Medicine, Einstein Medical Center Philadelphia, Philadephia, USA; 4 Neurology, SSM Health St. Mary's Hospital, St. Louis, USA

**Keywords:** cefepime, non-convulsive status epilepticus, neurotoxicity, seizure, encephalopathy, cefepime-induced seizures, nsce, drug-induced seizures, cefepime induced neurotoxicity

## Abstract

Cefepime, a widely used fourth-generation cephalosporin for coverage of both gram-positive and gram-negative bacteria, has been reported to have associated neurological adverse effects. These effects have been seen mostly in patients mostly with impaired renal function, and currently, dosing is based on creatinine clearance to reduce its toxic effect profile. Despite renal dose adjustment, we present a case of a 40-year-old woman who was managed for *Escherichia coli* bacteremia, acute kidney injury, and hemorrhagic shock. About 96 hours after cefepime therapy was commenced, she was noted to be twitching with passive movement of her upper limb and myoclonus of the facial muscles. Her workup including computed tomography (CT) scan of the head and magnetic resonance imaging (MRI) brain were negative. Electroencephalograph (EEG) showed 2 Hertz sharply contoured triphasic form rhythmic waves suggestive of nonconvulsive status epilepticus (NCSE). She received antiseizure medications and later had hemodialysis for effective clearance of cefepime. She had significant improvement in her neurological status following hemodialysis and a repeat EEG showed no further seizure activity. Clinicians should be aware of the risk of NCSE in patients on cefepime despite renal dose adjustment. Once identified, immediate discontinuation of the offending drug, treatment with benzodiazepines, and clearance of the medication with hemodialysis is recommended.

## Introduction

Cefepime is a widely used fourth-generation cephalosporin, especially in critical care units, because of its broad coverage of gram-positive and gram-negative bacteria, including *Pseudomonas* and *Klebsiella* [1-2]. There have been several reports of neurological adverse effects associated with cefepime including encephalopathy, seizures, and coma [3-5], with the first case reported in 1999 [6]. These adverse effects have been reported mostly in elderly patients and those with poor renal function [7-9]. In 2012, the FDA released a safety warning on the risk of seizures with the use of cefepime without renal adjustment [10]. We report a case of cefepime-induced non-convulsive status epilepticus (NCSE) in a young patient despite receiving a renal-adjusted dose of cefepime. 

## Case presentation

**Figure 1:**
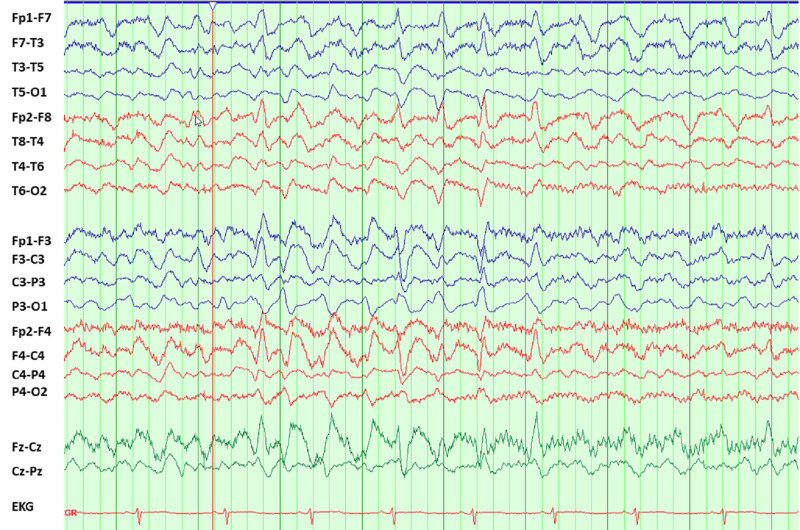
EEG showing diffuse slowing and triphasic waves EEG: Electroencephalograph

**Figure 2:**
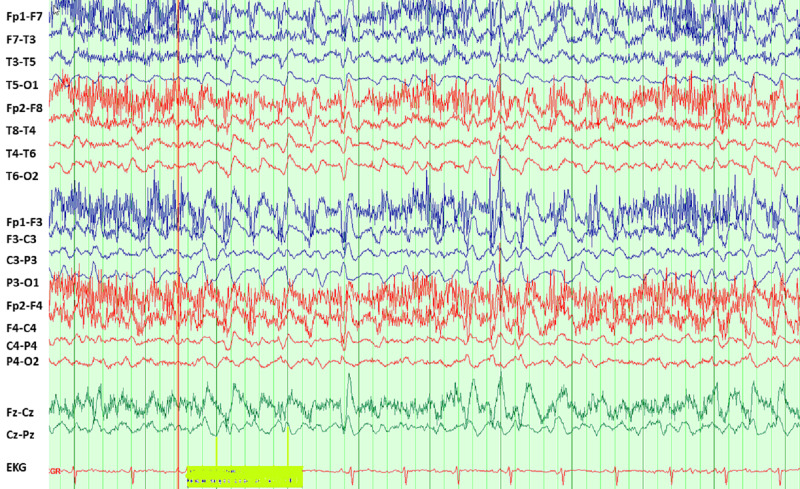
EEG showing sharply contoured triphasic form rhythmic waves suggestive of NCSE EEG: Electroencephalograph NCSE: Non-Convulsive status epilepticus

We present a case of a 40-year-old female who presented to the emergency department for nausea, vomiting, diarrhea, and left-sided abdominal pain. On admission, her temperature was 98.1 F (36.7 degrees C), with a blood pressure of 176/119 mmHg, heart rate of 111 bpm, tachypneic at 38 cpm, saturating 100% on room air. Initial laboratory workup showed leukocytosis of 15.2 K/uL (normal: 3.3 -8.7 K/uL) with 81% neutrophils (normal 44-73%), anemia of 10 (normal: 14-17 g/dL), thrombocytopenia of 134 K/uL (normal: 147-347 K/uL), elevated blood urea nitrogen (BUN) - 109 mg/dL (normal: 8-20 mg/dL), bicarbonate - 8 mmol/L (normal: 23-31 mmol/L), anion gap of 20 mmol/L (normal: 8-16 mmol/L), creatinine - 5.1 mg/dL (normal: 0.7-1.3 mg/dL), sodium - 139 mmol/L (normal: 136-145 mmol/L), potassium 4.8 mmol/L (normal: 3.5-5.1 mmol/L). Urinalysis showed 2+ protein, 3+ leucocyte, and negative nitrite. Urine microscopy showed >100 white blood cells (WBC), 51-100 red blood cells (RBC), and trace bacteriuria. She was started on empirical antibiotics with ceftriaxone for pyelonephritis. After 24 hours, hemoglobin dropped to 3.6 and she was admitted to the intensive care unit (ICU) for hemorrhagic shock secondary to upper gastrointestinal bleed for pressor support and acute hypoxic respiratory failure requiring intubation. She was started on hemodialysis for her acute kidney injury and later continuous renal replacement therapy (CRRT) due to shock. Ceftriaxone was escalated to cefepime 48 hours after initiation for worsening leukocytosis and preliminary blood cultures with gram-negative bacilli. She received 2 g every 12 hours of cefepime (based on dosing in hemofiltration). Urine culture later grew *Escherichia coli*. Upper limb extremity twitching was noted with passive movement of her upper limbs 96 hours after the commencement of cefepime. Neurology was consulted. MRI was negative for any acute intracranial process. Spot electroencephalograph (EEG) showed diffuse slowing suggestive of encephalopathy, triphasic waves with no evidence of epileptiform discharges or electrographic seizures (Figure 1). Due to continued twitching involving the face and bilateral upper extremities, EEG was converted to continuous which later showed 2 Hertz sharply contoured triphasic form rhythmic wave suggestive of non-convulsive status epilepticus (Figure 2). The discharges had clinical correlation with eye movements seen on the video EEG. Cefepime was discontinued and deescalated to ceftriaxone. The patient received lorazepam, loaded with levetiracetam, and started on propofol. She was later changed to midazolam drip due to concern for cefepime-induced seizures and its action on gamma-aminobutyric acid (GABA) receptors. Hemodialysis (HD) was completed to aid cefepime clearance. Following dialysis, her twitching stopped and no further electrographic seizures or triphasic waves were seen on video EEG. EEG was discontinued. The patient was weaned off sedation with improvement in neurological status. She was extubated three days after HD. Her renal function improved, and she was subsequently discharged home. 

## Discussion

Neurotoxicity has been reported with cephalosporins, especially cefepime, with varying clinical presentations including altered mental status, confusion, encephalopathy, seizures, asterixis, and coma [3-5,11]. The incidence of cefepime-related neurotoxicity accounts for about 1-3% and a systemic review reported a single-center estimate of one in 480 cases [12]. NCSE, one of the neurotoxic adverse effects of cefepime, encompasses a large group of different subtypes and currently does not have well-established diagnostic criteria. NCSE accounts for up to 25-50% of status epilepticus and can be fatal if left untreated [11,13]. They can occur as early as one day following the use of cefepime with a median time of onset of four days. Resolution of symptoms can be seen as early as two days after discontinuation of the medication [3]. This timing is similar to our patient’s presentation who developed NCSE within four days of commencement, and with the resolution of seizures seen within two days of drug discontinuation and treatment with hemodialysis.

The mechanism for NCSE in cefepime is not fully understood but has been described to be related to the increased penetration across the blood-brain barrier leading to inhibition of the GABA A receptors and a decrease in the seizure threshold [14]. With this mechanism, benzodiazepines remain the first-line medication for treatment due to their direct action on GABA receptors. NCSE can present with different EEG findings, including generalized periodic discharges, triphasic waves, generalized rhythmic delta activity, and spike waves [15]. EEG findings in our patient showed sharply contoured triphasic form rhythmic waves similar to EEG findings reported in cefepime-induced NCSE. 

The prevalence of NCSE is more common in elderly patients, female gender, and those with a history of epilepsy [16]. Another major risk factor for NSCE that has also been reported is impaired kidney function as cefepime is being cleared by the kidneys [9,12], necessitating renal-adjusted dosing. Our case is an example of NCSE in a patient receiving the right dose adjustment based on her creatinine clearance. This suggests that cefepime-induced NCSE can also occur in patients on the renal-adjusted dose, which has been reported in a few case reports [4,17-18]. It, therefore, requires more attention and a cautious approach when starting this medication in patients with any form of impaired renal function. The use of hemodialysis for effective clearance of the drug has been reported and when completed in our patient, EEG showed no further seizure findings, and she was easily weaned off sedation.

A high index of suspicion and awareness is essential as physicians. Unfortunately, monitoring drug levels are not widely available for cefepime. This can be an effective objective measure in determining patients that are high risk and might be a better form of dose adjustment compared to the current use based on creatinine clearance. In a recent study, cefepime trough plasma concentration >=36mg/L was seen to differentiate those who developed the neurotoxic side effects compared to those who did not [19], similar to findings by Huwyler et al. where neurotoxicity was associated with levels >35 mg/L [20].

## Conclusions

In conclusion, cefepime-induced NCSE still occurs in patients on renal adjusted doses. With the widespread use of cefepime especially in critically ill patients, close monitoring of the neurological status of these patients is highly recommended and if available, regular monitoring of the cefepime plasma concentration might help with early identification of patients at increased risk. Once identified, immediate discontinuation of the drug is essential, and in patients with no improvement, early clearance with hemodialysis can help to improve neurological status.
